# Comprehensive transcriptomic profiling and mutational landscape of primary gastric linitis plastica

**DOI:** 10.1007/s10120-022-01353-2

**Published:** 2022-11-30

**Authors:** Zhu Liu, Lian-Lian Hong, Jin-Sen Zheng, Zhe-Nan Ling, Zhi-Long Zhang, Ya-Nan Qi, Xin-Yu Zhang, Tian-Yu Zhu, Jiu-Li Wang, Jing Han, Xiang-Liu Chen, Qi-Ming Yu, Shi Wang, Pei Li, Zhi-Qiang Ling

**Affiliations:** 1 Zhejiang Cancer Institute, Institute of Cancer and Basic Medicine (ICBM), Chinese Academy of Sciences, Cancer Hospital of the University of Chinese Academy of Sciences, No.1 Banshan East Rd., Gongshu District, Hangzhou, 310022 People’s Republic of China; 2 Division of Hepatobiliary and Pancreatic Surgery, Department of Surgery, The First Affiliated Hospital, Zhejiang University School of Medicine, Hangzhou, Zhejiang Province People’s Republic of China; 3 Department of Oncology, The Second Clinical Medical College of Zhejiang, Chinese Medicine University, Hangzhou, Zhejiang Province People’s Republic of China; 4 Department of Pathophysiology, School of Basic Medical Sciences, Zhengzhou University, Zhengzhou, China; 5 Department of Digestive Oncology, The First Affiliated Hospital of Wenzhou Medical University, The First Provincial Wenzhou Hospital of Zhejiang, Wenzhou, 325000 China; 6 Department of Gastric Surgery, Institute of Cancer and Basic Medicine (ICBM), Chinese Academy of Sciences, Cancer Hospital of the University of Chinese Academy of Sciences, No.1 Banshan East Rd., Gongshu District, Hangzhou, 310022 People’s Republic of China; 7 Department of Endoscopy, Cancer Hospital of the University of Chinese Academy of Sciences (Zhejiang Cancer Hospital), Institute of Cancer and Basic Medicine (IBMC), Chinese Academy of Sciences, Hangzhou, China

**Keywords:** Gastric cancer (GC), Gastric linitis plastica (GLP), Scirrhous gastric cancer (SGC), Whole exome sequencing (WES), Whole transcriptome sequencing (WTS)

## Abstract

**Background:**

Primary gastric linitis plastica (GLP) is a distinct phenotype of gastric cancer with poor survival. Comprehensive molecular profiles and putative therapeutic targets of GLP remain undetermined.

**Methods:**

We subjected 10 tumor-normal tissue pairs to whole exome sequencing (WES) and whole transcriptome sequencing (WTS). 10 tumor samples were all GLP which involves 100% of the gastric wall macroscopically. TCGA data were compared to generate the top mutated genes and the overexpressed genes in GLP.

**Results:**

Our results reveal that GLP has distinctive genomic and transcriptomic features, dysfunction in the Hippo pathway is likely to be a key step during GLP development. 6 genes were identified as significantly highly mutated genes in GLP, including AOX1, ANKRD36C, CPXM1, PTPN14, RPAP1, and DCDC1). MUC6, as a previously identified gastric cancer driver gene, has a high mutation rate (20%) in GLP. 20% of patients in our GLP cohort had CDH1 mutations, while none had RHOA mutations. GLP exhibits high immunodeficiency and low AMPK pathway activity. Our WTS results showed that 3 PI3K-AKT pathway-related genes (PIK3R2, AKT3, and IGF1) were significantly up-regulated in GLP. Two genes were identified using immunohistochemistry (IHC), IGF2BP3 and MUC16, which specifically expressed in diffuse-type-related gastric cancer cell lines, and its knockdown inhibits PI3K-AKT pathway activity.

**Conclusions:**

We provide the first integrative genomic and transcriptomic profiles of GLP, which may facilitate its diagnosis, prognosis, and treatment.

**Supplementary Information:**

The online version contains supplementary material available at 10.1007/s10120-022-01353-2.

## Introduction

Gastric cancer (GC) is the fifth most common human malignancy and the third leading cause of cancer-related mortality worldwide [1]. GC classification is done based on different tissue structures, and the two most popular classification systems are Laurén classification and WHO classification [2]. Laurén classification of GC has always been the most commonly used classification system for GC. Laurén divided GC histology into two types, i. e., intestinal-type and diffuse-type [3]. Meanwhile, indeterminate-type was used to describe an uncommon histology [3–5]. Intestinal-type is the most common in GC, followed by diffuse-type and indeterminate-type [3]. Numerous studies have confirmed that intestinal-type GC is associated with intestinal metaplasia and helicobacter pylori infection in gastric mucosa, while diffuse-type GC mostly occurs in young female patients, which may indicate distinct tumor development pathways for intestinal-type and diffuse-type GC [6–8]. World Health Organization (WHO) classification includes not only gastric adenocarcinomas, but also all other types of gastric tumors with a low frequency. Gastric adenocarcinoma types fall into papillary, tubular, mucinous, and mixed cancers, which can be compared to indeterminate-type in Laurén classification [9–12]. Among the different subtypes, primary gastric linitis plastica (GLP) is a distinct phenotype of GC. Macroscopically, GLP is characterized by gastric wall thickening and a marked spread of the tumor to the submucosa and muscle layers. Microscopically, GLP is generally associated with signet ring cell gastric carcinoma features, diffuse and scirrhous histologic types [13–15].

The term “Scirrhous GC (SGC)”, a subtype of diffuse-type gastric cancer (DGC), is characterized by the macroscopic Borrmann type 4 or large (≥ 8 cm in diameter) Borrmann type 3 GC and by the microscopic undifferentiated cancer cells infiltration accompanied with extensive stromal fibrosis [13, 16]. Despite these particular features, there is no clear definition of GLP to date. GLP lacks a clear and standardized codification. “Linitis plastica” is used interchangeably with “Bolman type 4 carcinoma”, “scirrhous carcinoma,” “signet-ring cell carcinoma,” and “Lauren diffuse carcinoma”. However, it is not clear whether these terms correctly define the condition, as only some of the tumors in each of these categories have the features of GLP. [17–19]. GLP exhibits biological aggressiveness, including rapid infiltration into the gastric wall, progressive invasion into the serosa membrane, and seeding into the peritoneum, resulting in peritoneal metastasis [16]. GLP has a low incidence but a high mortality rate, their median overall survival (OS) duration ranges from 6 to 14 months [13]. The poor prognosis of GLP patients is due in part to the fact that the majority of GLP patients are not diagnosed at an early stage, and the rapid growth and invasion of this cancer makes clinical treatment challenging [20, 21]. There is no special treatment strategy for GLP, and it clinically follows the treatment means of general gastric cancer. A GLP definition based on molecular or genomic criteria could provide a new strategy for exploring potential targeted therapies.

The molecular mechanism underlying the development of GLP is unclear, and one reason is that the number of cases is too rare. With the large number of SGC cases, there are some related studies. Studies of SGC show that cytokines such as FGF, TGF, and HGF can promote SGC development and promote crosstalk between cancer cells and stromal fibroblasts [22]. Furthermore, the genetic changes in TP53 and CDH1 are thought to be related to SGC [23]. Mutations in RHOA and KMT2C were recently reported to be associated with SGC [24]. However, after all, SGC is not the same as GLP, and the understanding of the biological characteristics of GLP must be studied in cases truly defined as GLP [24, 25]. In this study, we first rigorously defined the primary GLP, and have collected 13 primary GLP specimens over the past 10 years. Using whole exome sequencing and transcriptome sequencing techniques, we analyzed the comprehensive transcriptome and mutational landscape of primary GLP, which provides insights into the biology of GLP, compensates for the lack of clinically actionable targets [26] and contributes to the accurate diagnosis and therapeutic development of GLP.

## Material and methods

### Definition of primary GLP and pathological analysis

Diagnostic criteria for the primary GLP as defined in this study [27]: (1) Macroscopic examination of the surgical specimens showed segmental or thickening of the gastric wall, with a lack of distensibility and sclerosis, and almost involving the whole stomach. (2) Histological examination revealed abundant and diffuse fibrous stromal reaction extended throughout the gastric lining to the subserosa. (3) Histological examination revealed more than 50% poorly cohesive cells having classical SRC morphology.

The exclusion criteria were as follows in this study [27]: hereditary gastric cancer, history of gastric surgery for any cause, history of endoscopic resection for superficial tumor prior to surgery (endoscopic mucosal resection or submucosal dissection), gastroesophageal junction cancer, non-adenocarcinomatous gastric tumor, adenocarcinoma infiltration of extra-gastric origin and secondary GLP. Secondary GLP, caused by metastasis of other tumors to the stomach, is most common in gastric metastases occurring in breast, lung, and pancreatic cancers.

### Tissue specimen

The study was approved by the Institutional Review Board of the University Cancer Hospital of the Chinese Academy of Sciences (IRB-2022–322). The patient and his or her family signed an informed consent form. Thirteen patients with primary GLP were first diagnosed by pathology at Cancer Hospital of the University of Chinese Academy of Sciences (Hangzhou, China) between January 2009 and December 2017 and without preoperative radiotherapy and chemotherapy or other tumors, and had complete clinical data. GLP tissues were obtained from the cancer nest and normal adjacent tissue from pathologically confirmed tissues 5 cm from the edge of tumors. These tissues were surgically removed and briefly stored in liquid nitrogen, and then transferred to a – 80 ℃ refrigerator for storage. We simultaneously collected peripheral blood leukocytes from all patients. Staging was performed according to the 7th edition International Cancer Alliance (UICC)/American Joint Committee on Cancer (AJCC) guidelines. Survival analysis was performed using overall survival (OS), defined as the time from surgery to death. All recruited patients had been followed up periodically until the due date.

### Data collection

The RNA expression data, somatic mutation data, and corresponding clinicopathological information of gastric cancer (TCGA-STAD) were obtained from the Genomic Data Commons (available at: https://portal.gdc.cancer.gov) Data Portal in December 21 2021. Samples without sufficient clinicopathological information or with pathological information of mixed subtypes were excluded from further analysis.

### Whole exome sequencing (WES)

The DNA from frozen samples was extracted using the QIAamp DNA Micro Kit (Qiagen, Stanford, CA, USA) according to the manufacturer's protocol. DNA was sheared into \ ~ 180–280 bp fragments using the Covaris S2 ultrasonic system (Covaris, Applied Biosystems, Carlsbad, CA, USA). Exome regions were captured using SureSelect All Exome V6 (Agilent, Santa Clara, CA, USA) according to the manufacturer's protocol. Then, the resultant products were paired-end sequenced using either an Illumina Hiseq Xten or a Novaseq 6000 system (5200 Illumina Way, San Diego, CA 92122, USA).

### Whole transcriptome sequencing (WTS)

Total RNA was isolated using RNeasy mini kit (Qiagen, Stanford, CA, USA). Paired-end libraries were synthesized using the TruSeq™ RNA Sample Preparation Kit (5200 Illumina Way, San Diego, CA 92122, USA) following TruSeq™ RNA Sample Preparation Guide. Briefly, the poly-A containing mRNA molecules were purified using poly-T oligo-attached magnetic beads. Following purification, the mRNA is fragmented into small pieces using divalent cations. The cleaved RNA fragments are copied into first strand cDNA using reverse transcriptase and random primers. This is followed by second strand cDNA synthesis using DNA Polymerase I and RNase H. These cDNA fragments then go through an end repair process, the addition of a single ‘A’ base, and then ligation of the adapters. The products are then purified and enriched with PCR to create the final cDNA library. Purified libraries were quantified by Qubit^®^ 2.0 Fluorometer (Life Technologies, Carlsbad, CA, USA) and validated by Agilent 2100 bioanalyzer (Agilent, Santa Clara, CA, USA) to confirm the insert size and calculate the mole concentration. Cluster was generated by cBot with the library diluted to 10 pM and then were sequenced on the Illumina NovaSeq 6000 (5200 Illumina Way, San Diego, CA 92122, USA).

### Molecular classification

Pre-defined gene signatures associated with gastric cancer molecular classification were applied for our analysis, which include EMT signature [28] and EBV signature [28]. R package Singscore (version 1.16.0) [29] was used to generate the corresponding EMT and EBV scores. MSI status were evaluated using MSIsensor [30]. CNVkit [31] was used for calling somatic copy number variants (CNV) to measure overall, arm and gene level CNV. The program’s default parameters were used to calculate the CNV-high and CNV-low cutoff values. GISTIC2 [32] were used to identify recurrent copy number variations across the cohort.

### Mutational signature analysis

Mutational signatures of our cohort were analyzed using the non-negative matrix factorization (NMF) method implemented in the R package maftools (version 2.10.0) [33]. We used cosine similarity as a metric to compare the similarities of our signatures to known signatures in COSMIC SBS signatures (v3.2).

### Differential expression analysis

Differential expression analysis for mRNA was performed using R package edgeR (version 2.10.0) [34]. Differentially expressed RNAs with |log2(FC)| value > 1 and *q* value < 0.05, considered as significantly modulated, were retained for further analysis.

### Hierarchical clustering of highly variable genes (HVGs)

For clustering among GLP, DGC, and GC, we first log-transformed the normalized expression matrix and then calculated the standard deviation (SD) for each gene across samples. The top 2000 genes with the highest SDs were selected and Spearman correlation was used as the distance metric for clustering. The analysis was performed with default parameters using pheatmap (version 1.0.12).

### Pathway enrichment analysis

The KEGG analysis on differentially expressed genes was performed using clusterProfiler (version 4.2.0) in R [35].

### Gene set enrichment analysis (GSEA)

GSEA analysis was performed using several pre-defined gene sets downloaded from the GSEA molecular signature database (MSigDb, http://software.broadinstitute.org/gsea/msigdb/index.jsp). Gene expression matrix was input and default parameters were used with *P* = 0.05 set as the cut-off to identify significant pathways.

### Cell culture, siRNAs, and transfection

GES1, AGS, NUGC4, and KATO III cells were obtained from ATCC. GES1, NUGC4, and KATO III cells were cultured in 90% RPMI 1640 medium (Gibco; Thermo Fisher Scientific, Inc.) supplemented with 10% fetal bovine serum (FBS; Gibco; Thermo Fisher Scientific, Inc.). AGS cells were cultured in 90% DMEM medium (Gibco; Thermo Fisher Scientific, Inc.) supplemented with 10% FBS. Cell lines were maintained at 37 °C and 5% CO2 in a humidified atmosphere and the medium was refreshed every 24 h. The short interfering RNAs (siRNA) were designed and synthesized by GenePharma (Suzhou, China), and were transiently transfected into cells using Lipofectamine 2000 Transfection Reagent (Invitrogen, Carlsbad, CA, USA) according to the manufacturer's instructions. The sequences of the siRNAs are provided in Table S1.

### Extraction of RNA and real-time PCR (RT-PCR)

Total RNA is extracted according to the instructions using Trizol (Invitrogen, Carlsbad, CA, USA). The concentration and purity were determined by Nano Drop UV spectrophotometer (ND-1000, USA). Reverse transcriptase was performed with One Step PrimeScript cDNA Synthesis kit (Takara Bio Inc, Kusatsu, Shiga, Japan), and cDNA was obtained for subsequent Real time RT-PCR detection according to the instructions. Real time RT-PCR was used to detect the target gene expression on 7500 Real Time PCR System-ABI 7500 (Applied Biosystem) with SYBR Premix Ex Taq™ Perfect Real Time kit (Takara Bio Inc, Kusatsu, Shiga, Japan). The primer sequence for target genes is shown in Table S2. GAPDH was used as an internal control. All experiments were repeated at least three times independently.

### Immunohistochemical (IHC) analysis

Immunohistochemical staining was performed as described in previous studies [36]. After deparaffinization, the antigen was recovered in 0.01 M citrate buffer, and then the endogenous peroxidase activity was inactivated in methanol for 10 min in 3% hydrogen peroxide. Non-specific binding was blocked by incubation with phosphate-buffered saline (PBS) of 10% normal goat serum for 1 h at room temperature. Prior to the addition of the primary antibody, 10% normal goat serum was incubated in phosphate buffer (PBS) for 1 h at room temperature to block the non-specific binding reaction. The slides were incubated with primary antibodies against IGF2BP3 (Product #: PA5-120,830, 1:100 dilution) (Thermo Fisher Scientific, 168 Third Avenue Waltham, MA USA 02451) or MUC16 (Ab272333, 1:800 dilution) (Abcam, Discovery Drive, Cambridge Biomedical Campus, Hills Road, Cambridge, CB20AX, United Kingdom, UK) overnight at 4 °C followed by biotinylated goat anti-mouse immunoglobulin G (Sigma, Mo, USA) was added for 1 h at room temperature. Then, the streptavidin–biotin-peroxidase complex assays were performed. The peroxidase activity was obtained by incubation with 0.1% 3,3-diaminobenzidine (Sigma) in PBS containing 0.05% hydrogen peroxide for 5 min at room temperature. As described methods in our previous report, based on the frequency and intensity of staining, the scores of histochemical staining were determined by three independent clinical pathologists, and the inconsistent results were finally determined after discussion [37]. For IGF2BP3 and MUC16 evaluation, the intensity score were designated as 0 (none), 1 (weak), 2 (moderate), and 3 (strong), and the percentages of stained cells were categorized as follows: 0% (none), 1% (weak), 2% (moderate), and 3% (strong). The percentage score is recorded as 0 (5%), 1 (5–25%), 2 (26–50%), 3 (51–75%), and 4 (76–100%). Multiplying the intensity score by the percentage score generated the final score.

### Western blotting (WB) analysis

Western blotting analysis was performed using Immobilon-P polyvinylidene difluoride membranes (Millipore, 28820 Single Oak Drive, Temecula, California 92590, USA). The cell lysates were separated on sodium dodecyl sulfate–polyacrylamide gels (SDS PAGE) and western blotting analysis was performed with following antibodies against IGF2BP3 (Product #: 14,642–1-AP, 1:500 dilution) (Proteintech Group, Inc, USA), PI3K (bs-2067R, 1:1000 dilution) (Bioss, Beijing, China), p-PI3K (bs-5570R, 1:1000 dilution) (Bioss, Beijing, China), AKT (10,176–2-AP, 1:1000 dilution) (Proteintech Group, Inc, USA), p-AKT (66,444–1-Ig, 1:5000 dilution) (Proteintech Group, Inc, USA) or MUC16 (Ab205718, 1:500 dilution) (Abcam, Discovery Drive, Cambridge Biomedical Campus, Hills Road, Cambridge, CB20AX, United Kingdom, UK), respectively. Anti-alpha Tubulin antibody [DM1A]—Loading Control (Ab7291) at 1/1000 dilution (Abcam, Cambridge, United Kingdom, UK) and GAPDH (D16H11, 1:1000 dilution) (Cell Signaling Technology) was used as an internal control. The density of band was measured using the ChemiDoc™ XRS + System (1000 Alfred Nobel Drive, Hercules, California 94547, USA) equipping with Image-Pro Plus software and Epson color image scanner. The data were normalized to the Tubulin or GAPDH.

### Statistical analysis

All statistical analyses were performed in the R statistical environment (version 4.1.2). All statistical tests, unless indicated otherwise, were two-sided tests with default p value set to 0.05 as the cutoff to indicate statistical significance.

## Results

### Patient characteristics, surgical and adjuvant treatment

Thirteen patients were diagnosed with primary GLP, met inclusion and exclusion criteria and were included in this study. Patients with GLP predominantly had signet ring cells and poorly differentiated histologic types (Table 1 and Table S3). All 13 patients with GLP underwent total radical gastrectomy by abdominal approach. All patients showed positive cytology, peritoneal carcinoma, and advanced pathological disease stage. Based on the TNM staging system, 2 of 13 GLP patients (15.4%, 2/13) were diagnosed as stage IIIC, and 11 (84.6%, 11/13) were diagnosed as stage IV. No metastases were found in all 13 patients at the time of initial diagnosis, but 11 patients were found to have adjacent organ invasion or distant metastases during or after surgery. Three patients received neoadjuvant chemotherapy. Patient No.2 received neoadjuvant chemotherapy of ECF regimen for three cycles. Patient No.6 received FOLFOX regimen for three cycles, while patient No.13 was treated with three cycles of EOX regimen. Detailed treatment of 13 GLP patients are shown in Table S3.

**Table 1:** Clinicopathological characteristics of patients with GLP enrolled in this study

Patient ID	Gender	Age	Pathological diagnosis	Size	T stage	N stage	M stage	Clinical stage
1	M	67	Linitis plastica	9 × 9 × 2.5 cm	T4b	N3	M1	IV
2	F	61	Linitis plastica	16 × 14 × 1.2 cm	T4b	N3	M1	IV
3	M	63	Linitis plastica	22 × 12 × 1.7 cm	T4b	N3	M1	IV
4	F	52	Linitis plastica	21 × 13 × 1.8 cm	T4b	N3	M1	IV
5	M	62	Linitis plastica	17.5 × 7.5 × 1.7 cm	T4b	N3	M1	IV
6	M	33	Linitis Plastica	17.5 × 13 × 1.5 cm	T4b	N3	M1	IV
7	M	39	Linitis plastica	18 × 14 × 2 cm	T4b	N3	M0	IIIC
8	F	57	Linitis plastica	15 × 11 × 1.8 cm	T4b	N2	M1	IV
9	F	36	Linitis plastica	17 × 14 × 1 cm	T4b	N3	M1	IV
10	M	75	Linitis Plastica	16 × 12.5 × 1.2 cm	T4b	N3	M1	IV
11	M	68	Linitis plastica	14 × 12 × 1.7 cm	T4b	N3	M0	IIIC
12	F	52	Linitis plastica	15 × 11.5 × 1.5 cm	T4b	N3	M1	IV
13	F	48	Linitis plastica	17.5 × 17 × 1.4 cm	T4b	N3	M1	IV

To measure the patient outcome, we analyzed clinical data on overall survival (OS), defined as the time from the start of surgery to death. All recruited GLP patients were regularly followed up until due date. Clinical follow-up results showed that the longest OS of 13 GLP patients was 25 months and the shortest was 1 month, and the mean OS was 13.08 months (Table S3).

### Specimen collection, preservation and pathological diagnosis

**Fig. 1:**
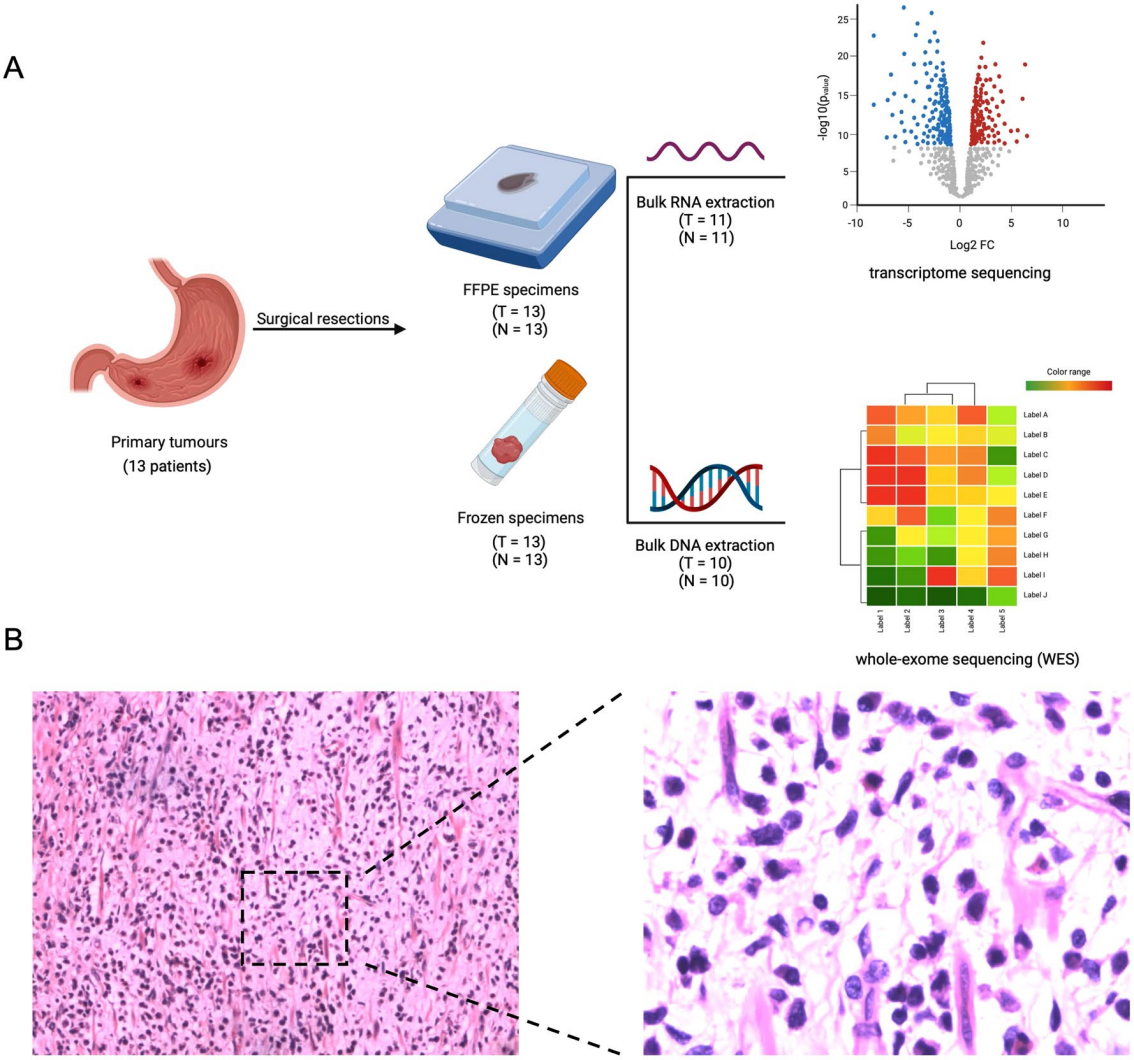
Research schematic **A** Methodology workflow. **B** Hematoxylin and eosin (H&E) staining of a typical gastric linitis plastica (GLP) sample. Scale bar, 400 μm, Left: × 10; Right × 400

We collected 13 surgically removed tumor samples and matched normal tissues from 13 GLP patients recruited from our hospital between 2009 and 2017. For three patients who received neoadjuvant therapy, we collected their pre-treatment gastroscopy samples for sequencing analysis. Fresh frozen tissues were sent for both whole exome sequencing (WES) and transcriptome sequencing (Fig. 1A). Formalin-fixed paraffin-embedded (FFPE) blocks were used for hematoxylin–eosin (H&E) staining for pathological diagnosis and subsequent immunohistochemical analysis. The histological review and the diagnosis of GLP were confirmed by three experienced pathologists based on H&E staining (Fig. 1B).

### Whole exome sequencing (WES)

**Fig. 2:**
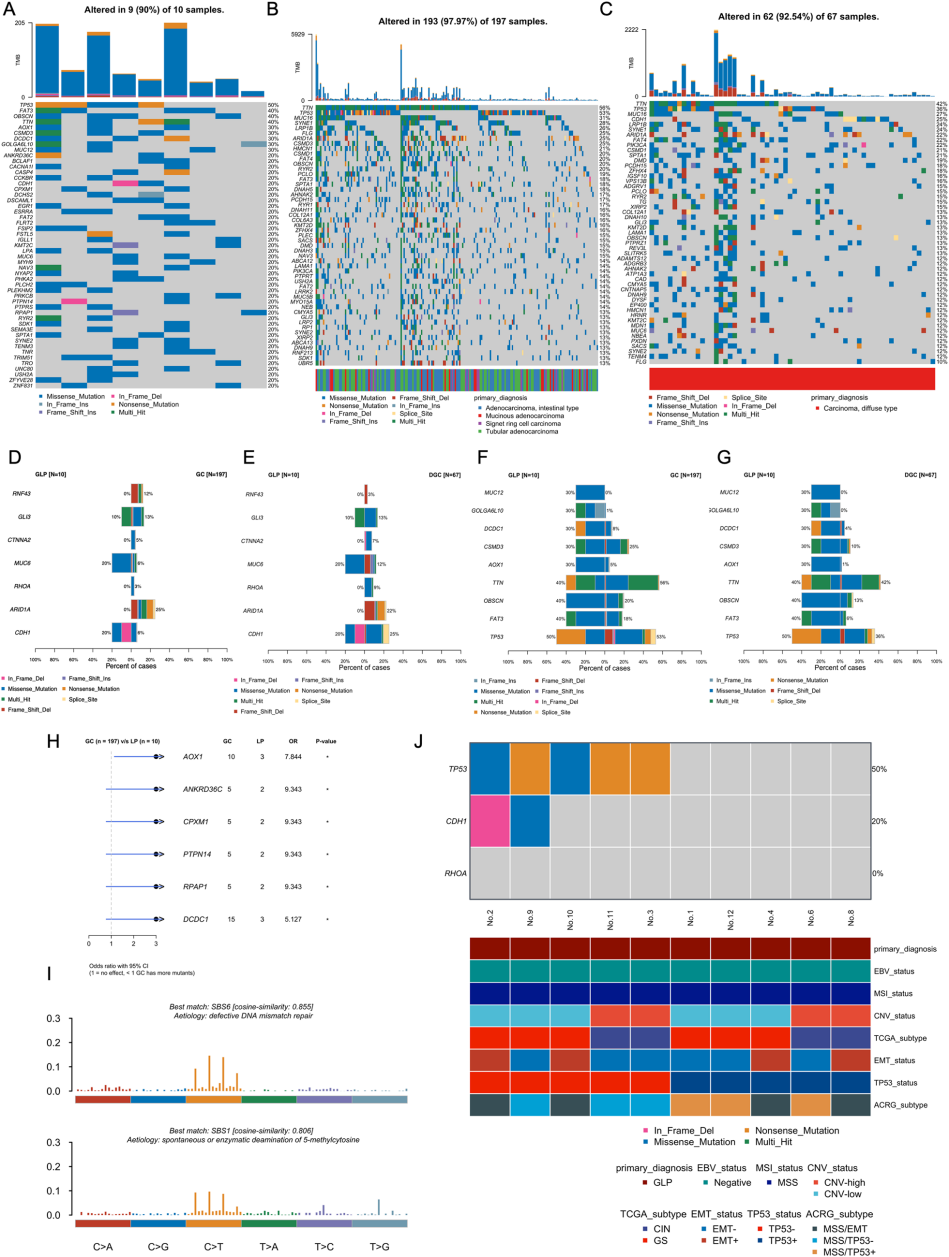
Somatic mutations analysis **A** Genomic landscape of GLP. The top bar plot shows the mutation burden for each sample. The body of the graph displays details about each gene, including different mutation types in different samples and the total mutation frequency across all patients with available WES samples. **B** Genomic landscape of GC without diffuse-type gastric cancer. The bottom of the graph shows the Pathological type of each sample. **C** Genomic landscape of DGC. **D** Comparison result of previously reported seven driver genes of gastric cancer between GLP and non-diffuse GC. Different colors indicated different mutation types. **E** Comparison result of previously reported seven driver genes of gastric cancer between GLP and DGC. **F** Comparison result of frequently mutated genes of GLP between GLP and GC. **G** Comparison result of frequently mutated genes of GLP between GLP and non-diffuse DGC. **H** Significantly differential mutated genes between GLP and GC, *P ≤ 0.05. Number of patients in each group, Odds ratio (OR), and p value are shown in the plot. **I** In each bar plot, the x-axis represented each one of the 96 types of the 3-bp sequence context and the y-axis indicated the frequency of the 96 substitution patterns. Mutational signatures were deciphered and cosine similarity was used as a metric to compare the similarity of deciphered signatures to known COSMIC SBS signatures. **G** TCGA and ACRG molecular subtypes of GLP patients, The top part of the graph shows the mutation status of TP53, CDH1, and RHOA, while the bottom part shows the information used for the molecular subtypes decision tree

The WES analysis was unsuccessful in 3 of the 13 samples due to inadequate DNA quality (Table S3). The global landscape of somatic mutations in GLP generated from WES data of 10 patients (10 GLP Tumor samples and matched blood or normal tissues as controls) is shown in Fig. 2A. The median depth of WES was 153.3X for tumors and 73.4X for normal controls (Table S4). A total of 1408 somatic changes were identified, including 1341 single nucleotide variations (SNVs) and 67 insertions or deletions (indels) (Table S5).

The most frequently mutated genes (> 20%) in our cohort are listed in Fig. 2A. Notably, the TP53 mutation was the most prominent variation, followed by FAT3, OBSCN, TTN, AOX1, CSMD3, DCDC1, GOLGA6L10, and MUC12. Then, we generated somatic mutations landscape using TCGA-STAD dataset. To better illustrate the mutation characteristics of GLP patients, we have excluded samples with diffuse-type gastric cancer and eventually, we got the mutation spectrum of 197 GC patients. The most frequently mutated genes generated from TCGA are shown in Fig. 2B. Different from the GLP landscape, TTN mutation was the most widespread mutation in GC patients, followed by TP53, MUC16, SYNE1, LRP1B, FLG, ARID1A, and CSMD3. Further, we have also generated the mutational spectrum of diffuse-type gastric cancer, as shown in Fig. 2C, the most frequently mutation was TTN, followed by TP53, MUC16, CDH1, LRP1B, SYNE1, and ARID1A.

To investigate the mutational patterns of GLP patients, we systematically compared their genomic features to those of patients with GC or DGC. First, we focused on well-known cancer genes related to GC tumorigenesis, genomic variations of seven driver genes of gastric cancer, including two genes closely related to diffuse-type gastric cancer reported in previous studies were compared between datasets (Fig. 2D, E) [38, 39]. CDH1, which encodes a classical cadherin of the cadherin superfamily E-cadherin, was mutated in 20% of GLP patients in our cohort, a frequency comparable to that of non-diffuse GC (6%) and DGC (25%), indicating the significance of CDH1 in pathological diffuse-type gastric cancer, including GLP and DGC. However, RHOA mutation—another diffuse-type gastric cancer-related genomic variation [40], which was mutated in 3% of non-diffuse GC patients and in 9% of DGC patients, was not detected in any GLP patients in our cohort. Regarding GC driver genes, 25% of non-diffuse GC and 22% of DGC had ARID1A mutations, but none of the patients in our GLP cohort bore ARID1A mutation. MUC6 mutation was more prevalent in GLP (20%) than in non-diffuse GC (6%) and DGC (12%).

Secondly, we looked at the most frequently mutated genes in GLP and compared them with non-diffuse GC and DGC cohort (Fig. 2F, G). TP53 and TTN are the most frequently mutated genes in three cohorts. Six genes (FAT3, OBSCN, AOX1, DCDC1, GOLGA6L10, and MUC12) show a disproportionate distribution of mutations when compared to non-diffuse GC and DGC, indicating that GLP has distinctive genomic features. To further analyze these genomic features, we calculated differential mutated genes from our WES data and TCGA non-diffuse GC data and found six genes were significantly highly mutated in GLP cohort (Fig. 2H). AOX1, a cancer-related gene encoded Aldehyde Oxidase 1 [41] and mutated in 30% of our GLP cohort, was found to play an oncogenic role in cancer progression through its epigenetic changes [42], but the relationship between its mutations and cancer have not been well reported. CPXM1, a putative tumor suppressor gene [43], was found to have a positive relationship with immunotherapy in head and neck squamous cell carcinoma [44]. PTPN14, encoded a member of the protein tyrosine phosphatase (PTP) family, its mutation was considered to be associated with cervical cancer and be a high-impact basal cell carcinoma predisposition gene [45]. DCDC1, encoded a member of the doublecortin family, was found to be a gene that significantly mutated in oesophageal squamous cell carcinoma [46]. Furthermore, we noticed that six genes mutated in our GLP cohort were identified as tumor suppressor genes in the Hippo pathway (Figure S1), including 2 GLP highly mutated genes (FAT3, and PTPN14). Considering the Hippo pathway plays a crucial role in cell proliferation as well as communication between cancer cells and stromal cells [47]. Dysfunction of the Hippo pathway might be a key step during the GLP development. Those observations indicated that compared to GC, GLP may have distinctive genomic features.

Somatic mutations in cancer genomes are caused by multiple mutational processes. Different mutational processes generate unique combinations of mutation types, we can analyze that “fingerprints” to get hints of cancer aetiologies. A non-negative matrix factorization (NMF) method was used to analyze the mutational signatures of GLP. We identified two prominent signatures (Fig. 2I), and cosine similarity was used to compare those signatures to known Single Base Substitution (SBS) signatures in COSMIC. The two signatures identified using our data were similar to COSMIC signature SBS6 (cosine similarity: 0.885) and SBS1 (cosine similarity: 0.806), respectively. COSMIC signature SBS6 is believed to be associated with defective DNA mismatch repair, while COSMIC signature SBS1 is believed to be primarily caused by the spontaneous or enzymatic deamination of 5-methylcytosine.

### Molecular subtypes analysis

According to The Cancer Genome Atlas (TCGA)[48] and Asian Cancer Research group (ACRG)[49] suggestion, we investigated the molecular subtypes of each case in our GLP cohort. The TCGA Working Group divided gastric cancer into four subtypes (EBV, MSI, genomically stable GS, and chrom instability CIN) based on molecular classification. Based on EBV scores generated from EBV signature[28] and WES data, all the GLP samples were evaluated as EBV-negative and MSS (Figure S2 and Table S6). CNV data indicated that 4 samples were classified to the CNV-high group, while 6 samples were classified to the CNV-low group (Figure S3 and Table S6). According to the TCGA decision tree, 4 patients were assigned to the CIN group and 6 were assigned to the GS group (Fig. 2J). Among these four categories, the characteristics of the GS group are histological diffuse-type and mutations of RHOA and CDH1 [48]. We observed that the majority of patients in our GLP cohort (6/10), similar to those reported by the TCGA working group, were categorized in the GS group and tended to have the CDH1 mutation (2/6), but no RHOA mutations were observed in the GLP cohort (Fig. 2J). In addition, ACRG molecular subtypes also divided gastric cancer into four subtypes (MSI, MSS/EMT, MSS/TP53, and MSS/TP53+)[49]. EMT scores were generated using the EMT signiture (Figure S4 and Table S7), and four samples were considered EMT+. We classified 4 samples into the MSS/EMT group, 3 samples into the MSS/TP53 + group, and 3 samples into the MSS/TP53- group based on the ACRG decision tree (Fig. 2J) (Table S7). According to ACRG research, diffuse gastric cancer has greater heterogeneity in ACRG subtypes compared to TCGA subtypes [49], which is consistent with our observations.

### Whole transcriptome sequencing (WTS)

**Fig. 3:**
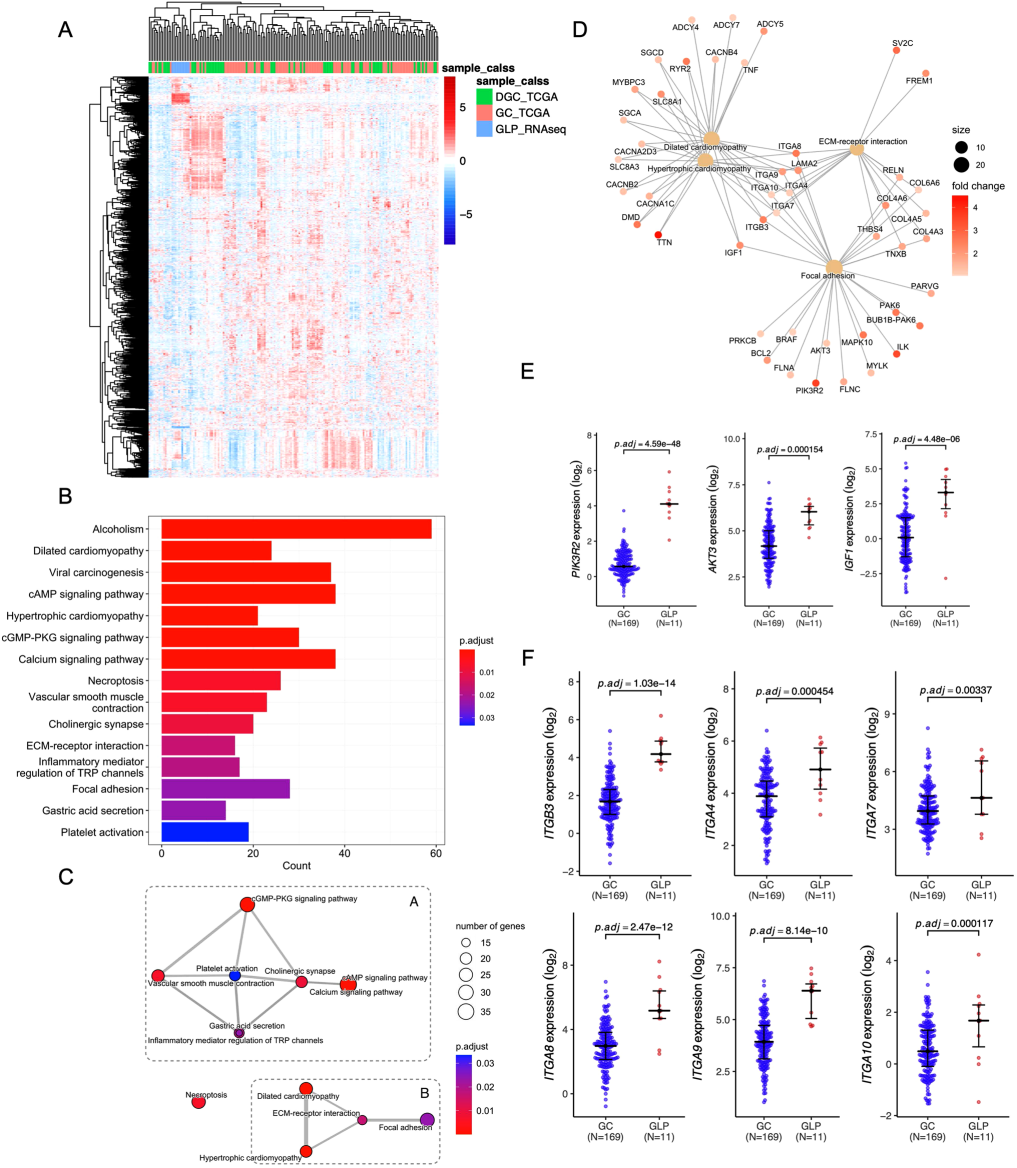
Transcriptomic analysis **A** Heat map of the hierarchical clustering of the top 2000 highly variable genes (HVGs) across GLP and GC datasets. The Spearman correlation coefficient was used as the distance metric for clustering. **B** Bar plot of Kyoto Encyclopedia of Genes and Genomes (KEGG) enrichment analysis showing the KEGG terms of genes up-regulated in GLP as compared to GC. **C** Two functional modules found from KEGG enrichment analysis. **D** Up-regulated genes in GLP-related to module B. **E**, **F** Genes that are differentially expressed between GLP and GC, p-adjust values (BH method) are shown in each result

The WTS analysis was unsuccessful in 2 of the 13 samples due to inadequate RNA quality (Table S3). We then studied the gene expression features of GLP. We First investigated how distinctive GLP is at the transcriptome level compared to GC. We downloaded mRNA expression data from the TCGA-STAD dataset and divided it into non-diffuse GC (169 cases) and diffuse-type DGC (61 cases) for further analysis. The results of hierarchical clustering of 2000 highly variable genes (HVGs) across our GLP samples and TCGA samples showed that GLP has its own distribution pattern at the transcriptome level (Fig. 3A). In addition, while some DGC patients showed an expression profile similar to GLP, some DGC samples were more comparable to GC, which supports our hypothesis that pathological diffuse Borrmann IV GC might be further separated into subtypes with a different tumorigenesis and development mechanism.

Next, Kyoto Encyclopedia of Genes and Genomes (KEGG) enrichment analysis were performed to explore the transcriptional differences between GLP and non-diffuse GC (Fig. 3B). We found two functional modules from KEGG pathway enrichment (Fig. 3C), the module B composed of four pathways (Dilated cardiomyopathy, Hypertrophic cardiomyopathy, ECM-receptor interaction, and Focal adhesion), which are obviously related to the extracellular matrix and have a high probability of contributing to the pathological stromal stiffness of GLP. Moreover, we investigated the genes involved in module B (Fig. 3D) and found several genes involved in the PI3K-AKT pathway were significantly up-regulated in the GLP cohort (Fig. 3E). Such as PIK3R2, a Key regulatory subunit of PI3K, which was considered an oncogene in several cancers [50]. AKT3, a central gene in the PI3K-AKT pathway. IGF1, upstream growth factor of PI3K-AKT pathway that was recently found to be related with the mesenchymal phenotype of gastric cancer [51]. In addition of that, a whole bunch of IGTA/IGTB Integrins superfamily members were found up-regulated in GLP (Fig. 3F), which are also upstream molecules of PI3K-AKT pathway and ITGB3, which encoded integrin beta chain beta-3, was considered a cancer-associated fibroblasts (CAFs)-related gene and induced breast tumor invasiveness through crosstalk between CAFs and tumor cells[52]. These results suggested that the PI3K-AKT axis, which has been already activated in gastric cancer [53], might be more strongly activated in GLP and perhaps play an important role in the pathological changes of GLP.

**Fig. 4:**
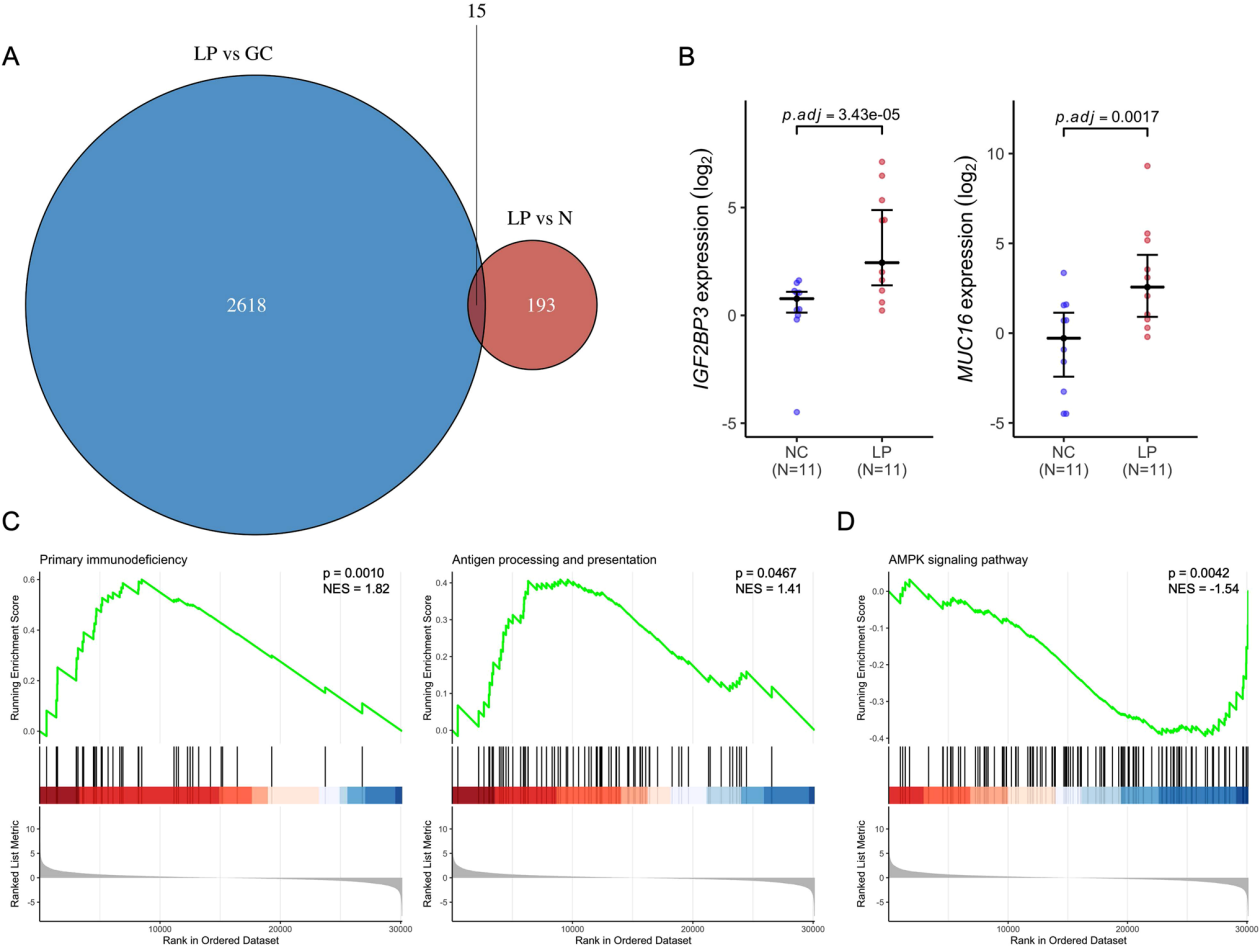
Potential targeting-genes in GLP. **A** Venn diagram for genes which up-regulated in GLP compared to both GC samples and normal samples. **B** IGF2BP3 and MUC16 expression between GLP and GC samples. **C** Gene Set Enrichment Analysis (GSEA) enrichment analysis between GLP and GC group. Normalized enrichment score (NES) and p values are shown in each result

Furthermore, we generated the DEGs significantly up-regulated in GLP by comparing expression data from GLP tumors and matched normal tissues (*P* < 0.05 and LogFC > 1). Then, the same procedure was performed based on our GLP cohort and TCGA-STAD non-diffuse GC cohort. Using the Venn diagram, we finally found 15 genes specifically up-regulated in GLP (Fig. 4A and Table S8). Two of these fifteen genes were reported to be closely associated with tumor development (Fig. 4B), which aroused our interest. As an RNA-binding protein associated with the PI3K pathway [54], IGF2BP3 can function as a m6a reader to engage in tumor m6a modification [55, 56]. Another gene, MUC16, also known as CA125, is the gold standard biomarker used to diagnose and monitor ovarian cancer progression and recurrence and has been reported to be overexpressed in several cancers [57]. Therefore, we selected these two genes for further analysis and verification of their role in GLP development. Both genes have been found to play a significant role in the tumor microenvironment associated with the diffuse phenotype [58, 59] and are overexpressed in our cohort. It is worth noting that MUC16 is a highly mutated gene (38.4%) in GC [60]. However, we did not find MUC16 mutations among our top mutated genes from WES data; instead, we observed a substantially up-regulated MUC16 expression level in GLP (Fig. 4B). The evidence found in other studies [61, 62], together with our findings, implies that IGF2BP3 and MUC16 might participate in the process of GLP development, and might be potential targets for strategies of GLP treatment.

### Pathway analysis

As we know, GLP patients tend to have an extremely poor prognosis, existing treatment strategies are not satisfactory for GLP patients. So, we performed a GSEA enrichment analysis to further reveal the potential valuable pathway changes of GLP. By comparison of all DEGs generated from GLP and matched normal tissues, we found the immune-related pathways significantly enriched in the GLP group (Fig. 4C). Immunotherapy has emerged as a breakthrough for therapy of gastric cancer [63]. The pathway of primary immunodeficiency (*P* = 0.0010) and of antigen processing and presentation (*P* = 0.0467) are both activated in GLP, which shows that the application of immunotherapy in GLP might be worth a try. additionally, the inactivation of the AMPK pathway in GLP (Fig. 4D) also suggested AMPK activators (e.g., Metformin) might be able to play a potential role in GLP treatment.

### MUC16 and IGF2BP3 are overexpressed in diffuse/signet ring gastric cancer cell lines and activate the PI3K-AKT pathway

**Fig. 5:**
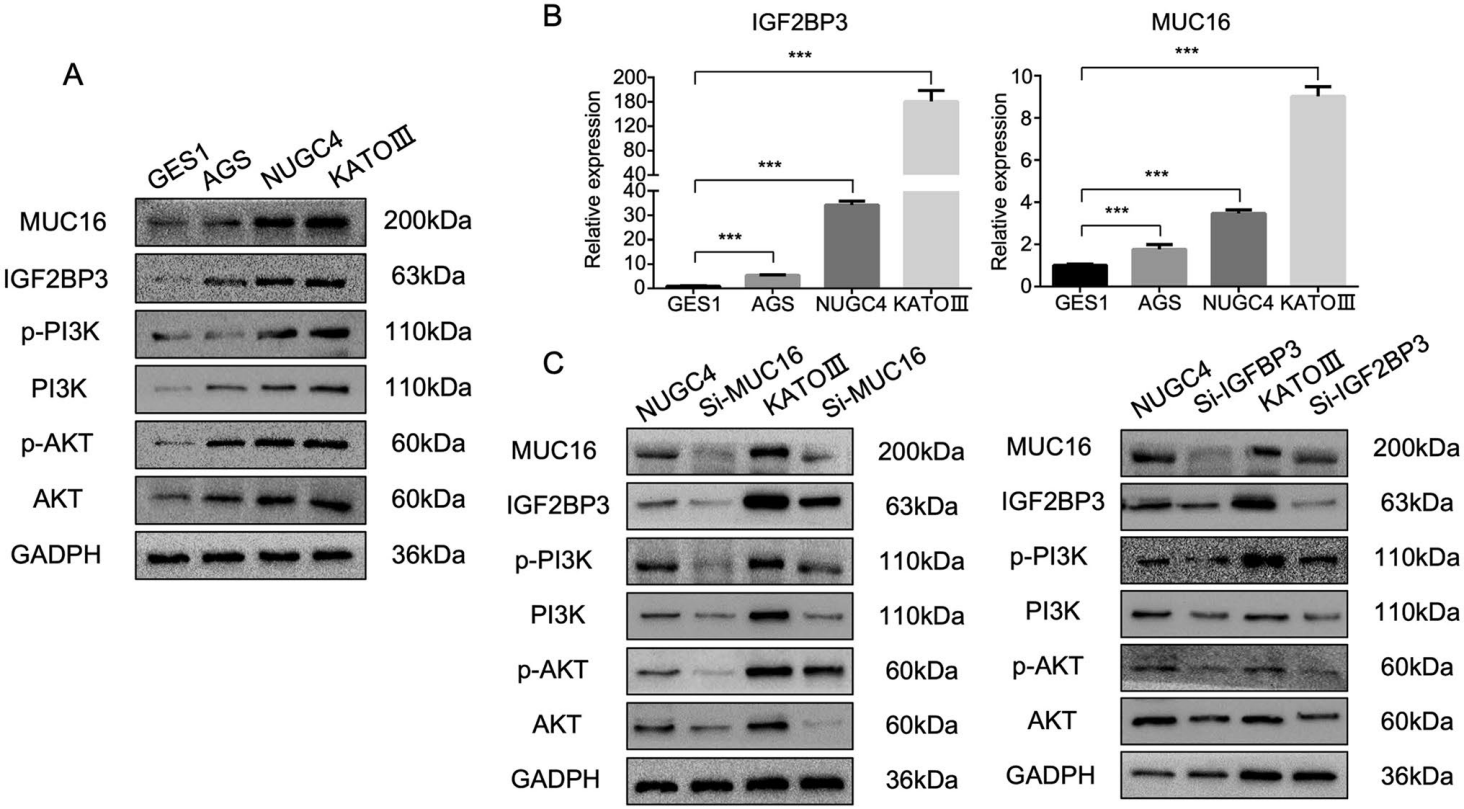
MUC16 and IGF2BP3 affect PI3K-AKT pathway activity. **A** Western blot result of targeted genes expression in GES1, AGS, NUGC4, and KATO III cell lines. **B** qRT-PCR results of IGF2BP3 and MUC16 in GES1, AGS, NUGC4, and KATO III cell lines. **C** Western blot result of targeted genes expression in NUGC4 and KATO III cell lines after MUC16 or IGF2BP3 knockdown using siRNAs

To validate the findings of WTS in GLP tissues, we analyzed the expression of IGF2BP3 and MUC16 in the human gastric epithelial cell line GES1 and the human gastric cancer cell lines AGS (intestinal-type), NUGC4 (diffuse-type), and KATO III (signet ring cell carcinoma, SRCC). As shown in Fig. 5A, IGF2BP3 and MUC16 are overexpressed in gastric cancer cell lines, and they are significantly upregulated in diffuse gastric cancer cell lines and SRCC cell lines that are closely related to diffuse gastric cancer[64, 65]. In addition, consistent with previous reports [54, 66], the PI3K-AKT pathway was activated in DGC/SRCC gastric cancer cell lines. Using qRT-PCR, we also observed IGF2BP3 and MUC16 mRNA upregulation in DGC/SRCC cell lines (Fig. 5B). Knockdown of IGF2BP3 and MUC16 in NUGC4 and KATO III cell lines using siRNA led to a significant decrease in p-PI3K and p-AKT (Fig. 5C and Figure S5), confirming its impact on PI3K-AKT pathway activation.

### IGF2BP3 and MUC16 expression detected by IHC and WB analysis

**Fig. 6:**
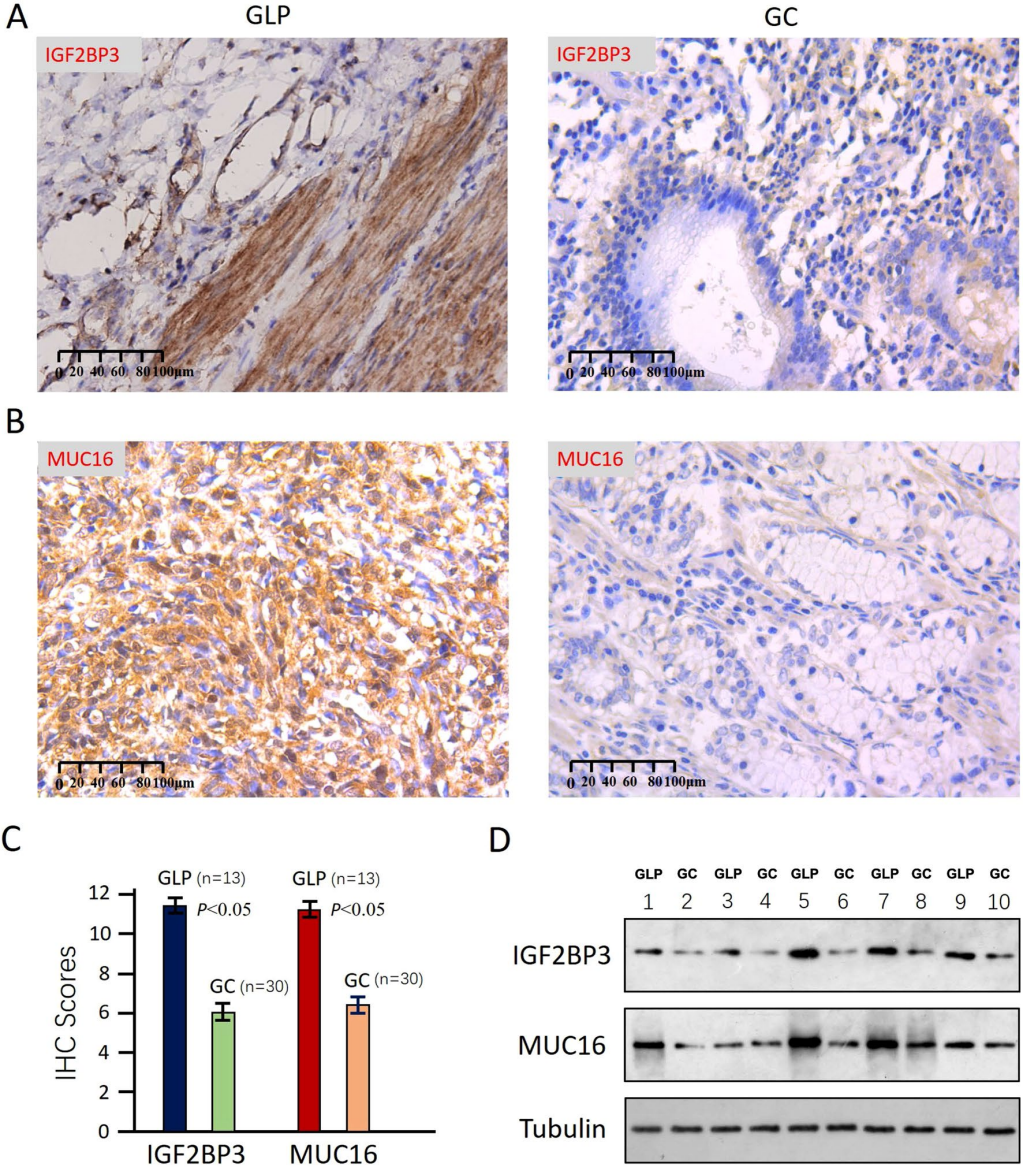
IGF2BP3 and MUC16 expression detected by IHC and WB analysis **A–C** Typical immunohistochemistry images and corresponding IHC scores of GLP and GC sample. **A** IGF2BP3 staining. **B** MUC16 staining. **C** Corresponding IHC scores. D Western blot result of IGF2BP3 and MUC16 expression levels in five GLP and five GC tissue samples, odd numbered for GLP samples and even numbered for GC samples

To validate the findings in human gastric cancer specimens, we first analyzed the expression levels of IGF2BP3 and MUC16 in 13 GLP and 30 gastric cancer tissues using immunohistochemistry. The clinical information of 30 GC cases was shown in Table S9. The results showed that IGF2BP3 and MUC16 were significantly upregulated in GLP tissues, as compared with GC tissues (Fig. 6A–C). Then, we used western blot to analyze IGF2BP3 and MUC16 expression levels in five GLP and five GC tissue samples, and showed that IGF2BP3 and MUC16 were significantly upregulated in GLP tissues compared with GC tissues. Both immunohistochemistry and WB analysis supported the finding of WTS in GLP, namely that IGF2BP3 and MUC16 expression were significantly upregulated in GLP tissues (Fig. 6D), suggesting that IGF2BP3 and MUC16 may play an important role in GLP development, and the precise mechanism needs further investigation.

## Discussion

GLP, a rare and aggressive subtype of GC, morphologically shows rapid infiltration in the gastric wall, progressive invasion into the serosal layer, and seeding to the peritoneum. Using the two classifications that have been used clinically, GLP can be defined as macroscopic Borrmann type 4 and Laurén diffuse-type. However, its molecular and pathological features have remained widely unexplored. According to the study from The Cancer Genome Atlas (TCGA) Research Network [48], the majority of GLP can be classified into the GS group defined by the TCGA classification, which is characterized by diffuse histology, mutations in CDH1/RHOA, and fusions in the CLDN18 family, despite there is insufficient data regarding this issue [48]. Although, in this study, the high frequency of RHOA mutation was not observed in our GLP cohort. The similarities of our GLP mutation signature and COSMIC signature SBS6 suggested that GLP might harbor genomic features related to the dysfunction of DNA mismatch repair. These observations indicated that pathological diffuse-type GC can further be divided into subtypes with different molecular features. GLP, as a typical subtype of diffuse-type gastric cancer, considering its extreme stiffness of the stomach wall and its unsatisfactory survival outcomes, might be a suitable target to explore the mechanism of such kind of GC.

We revealed the distinctive genomic features of GLP in this study. First, in the comparison of seven well-know driver genes of gastric cancer, the disproportionate distribution of the CDH1 mutation observed in our GLP cohort and the TCGA non-diffuse GC cohort further supports the previous studies about DGC [48, 67]. As we know, germline mutations in the tumor suppressor gene CDH1 could lead to hereditary diffuse gastric cancer (HDGC) [67], our finding about somatic mutations of CDH1 in GLP further emphasizes the importance of the CDH1 mutations in certain subtypes of gastric cancer. The absence of RHOA mutations in our GLP cohort also accords with the earlier observations that high frequency of somatic CDH1 alterations, but low frequency of somatic RHOA mutations were found in early onset diffuse gastric cancer [39]. In addition, we found the MUC6 another suspected driver gene in GLP for the first time and further work is required to explore the relationship and possible mechanism of MUC6 mutations in GLP. Second, the analysis about the most frequently mutated genes and the finding of dysfunction of the Hippo pathway in GLP indicated distinctive genomic features of GLP. Several recent studies had demonstrated the important role that the Hippo pathway plays in the pro-fibrotic process [68]. The Hippo pathway converges with pro-fibrotic signaling pathways, which can induce pro-fibrotic effects with or without TGFβ [69, 70], and establish and coordinate a fibrogenic signaling network [71]. Hence, it could conceivably be hypothesized that abnormal function of the Hippo pathway in GLP could possibly be a crucial step of progression of pathological scirrhous-type.

The WTS analyses revealed that GLP showed an overactivation of the PI3K-AKT pathway, which has already been found activated in common types of gastric cancer [53]. As we already know, Hippo/TGF-β/WNT/beta-catenin/PI3K/AKT generates a huge regulatory network that is involved in the progression of various cancers [72]. Together with our findings, it is therefore likely that such network exhibit an overactive state in GLP, and the key genes we found in that network might be valuable for further research. Recently, studies about IGF2BP3 showed the connection between the immunohistochemical evaluation of IGF2BP3 expression and tumor growth and prognosis [51, 73]. In hepatocellular carcinoma, the expression of IGF2BP3 is correlated with advanced tumor stage/grade and metastasis [74]. Moreover, MUC16 was widely reported as a frequently mutated gene in GC and was thought to be related to tumor mutation burden (TMB) [60]. However, instead of a frequently mutated gene, we found MUC16 an over-upregulated gene in GLP. The role it plays in cancer, especially in cancer with massive stroma stiffness still needs to be clarified in further research.

To some extent, the most important limitation lies in the fact that the limited sample size reduces the statistical significance and the generalities of our results. Future studies with larger sample size are required to further explore the molecular features of GLP. In addition, immunohistochemical validation suggested that IGF2BP3 staining appeared in fibroblasts surrounding the tumor tissue. Considering the correlation between CAFs and tumor fibrosis [75, 76], it remains to be determined whether IGF2BP3 expressed by fibroblasts has an effect on the biological behavior of tumor cells. Another limitation of this study is that, limited by the rarity of samples meeting the diagnostic criteria and the difficulty of early diagnosis, all samples were advanced GLP, which may have obscured the role of GLP-related genes, such as IGF2BP3 and MUC6, in the carcinogenesis and progression of GLP. Experiments aiming to examine the carcinogenesis of GLP using animal models are conceivable, and future research in this direction may be able to shed more light on the underlying mechanisms of GLP. In summary, we revealed the distinctive genomic features of GLP and found that GLP displays overactive Hippo/PI3K-AKT pathway activities, several key genes in that pathway might be new targets for GLP treatment. Finally, we verified the IGF2BP3 and MUC16 expression using Immunohistochemistry in GLP and GC patients. We provide valuable biological and clinical insights into this disease.

## Supplementary Information

Below is the link to the electronic supplementary material.

Supplementary file1 (XLSX 10 KB)

Supplementary file2 (XLSX 9 KB)

Supplementary file3 (XLSX 12 KB)

Supplementary file4 (XLSX 13 KB)

Supplementary file5 (XLSX 110 KB)

Supplementary file6 (XLSX 10 KB)

Supplementary file7 (XLSX 10 KB)

Supplementary file8 (XLSX 9 KB)

Supplementary file9 (XLSX 12 KB)

Supplementary file10 (DOCX 5866 KB)

## Data Availability

The data used to support the findings of this study are available from the corresponding author upon request.
