# Correction: Comprehensive transcriptomic profiling and mutational landscape of primary gastric linitis plastica

**DOI:** 10.1007/s10120-023-01383-4

**Published:** 2023-04-05

**Authors:** Zhu Liu, Lian-Lian Hong, Jin-Sen Zheng, Zhe-Nan Ling, Zhi-Long Zhang, Ya-Nan Qi, Xin-Yu Zhang, Tian-Yu Zhu, Jiu-Li Wang, Jing Han, Xiang-Liu Chen, Qi-Ming Yu, Shi Wang, Pei Li, Zhi-Qiang Ling

**Affiliations:** 1 Zhejiang Cancer Institute, Zhejiang Cancer Hospital, Institute of Cancer and Basic Medicine (ICBM), Chinese Academy of Sciences, No.1 Banshan East Rd., Gongshu District, Hangzhou, 310022 People’s Republic of China; 2 Division of Hepatobiliary and Pancreatic Surgery, Department of Surgery, The First Affiliated Hospital, Zhejiang University School of Medicine, Hangzhou, Zhejiang Province People’s Republic of China; 3 Department of Oncology, The Second Clinical Medical College of Zhejiang, Chinese Medicine University, Hangzhou, Zhejiang Province People’s Republic of China; 4 Department of Pathophysiology, School of Basic Medical Sciences, Zhengzhou University, Zhengzhou, China; 5 Department of Digestive Oncology, The First Affiliated Hospital of Wenzhou Medical University, The First Provincial Wenzhou Hospital of Zhejiang, Wenzhou, 325000 China; 6 Department of Gastric Surgery, Zhejiang Cancer Hospital, Institute of Cancer and Basic Medicine (ICBM), Chinese Academy of Sciences, No.1 Banshan East Rd., Gongshu District, Hangzhou, 310022 People’s Republic of China; 7 Department of Endoscopy, Zhejiang Cancer Hospital, Institute of Cancer and Basic Medicine (IBMC), Chinese Academy of Sciences, Hangzhou, China

**Correction: Gastric Cancer (2023) 26:203–219** https://doi.org/10.1007/s10120-022-01353-2

In the original publication of the article, the affiliations are wrongly published and should be corrected as given in this correction.

