# Supplementary material for: Comprehensive transcriptomic profiling and mutational landscape of primary gastric linitis plastica
Source: Gastric Cancer. 2022 Nov 30;26(2):203–19. doi: 10.1007/s10120-022-01353-2 (PMC9950178; doi:10.1007/s10120-022-01353-2)
Supplement: Supplementary file 10 — Supplementary file10 (DOCX 5866 KB) [file 10120_2022_1353_MOESM10_ESM.docx]

## Comprehensive transcriptomic proﬁling and mutational landscape of primary gastric linitis plastica

Zhu Liu^1,8^, Lian-Lian Hong^1,8^, Zhe-Nan Ling^2^, Zhi-Long Zhang^3^, Ya-Nan Qi^4^, Xin-Yu Zhang^3^, Tian-Yu Zhu^5^, Jiu-Li Wang^1^, Jing Han^1^, Xiang-Liu Chen^1,6^, Qi-Ming Yu^6^, Shi Wang^7^, Pei Li^4^, Zhi-Qiang Ling^1^*

## Supplementary Figure.


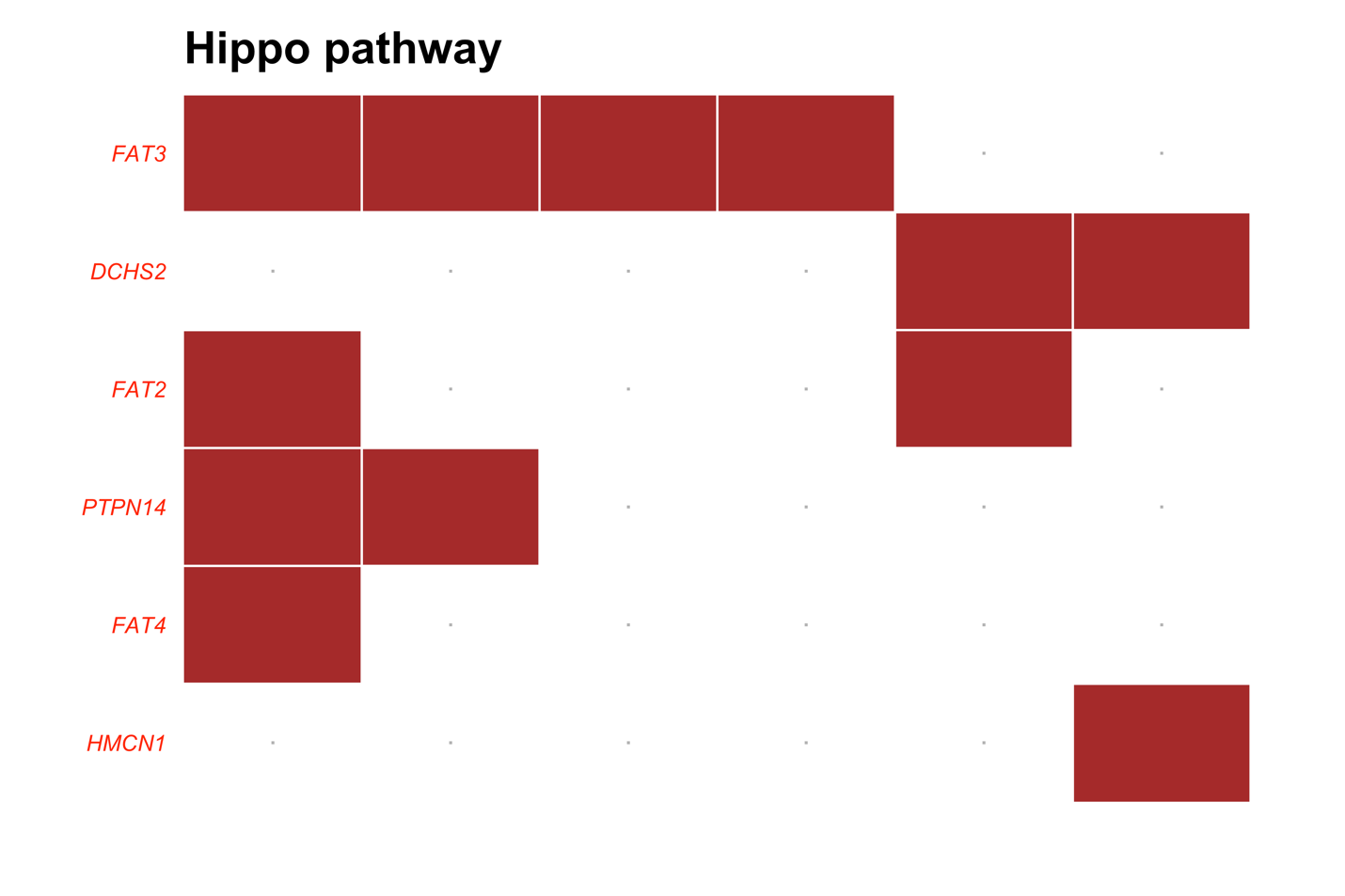


**Figure S1. Tumor suppressor genes in the Hippo pathway**. Mutation pattern of six tumor suppressor genes in the Hippo pathway, the x-axis represented each one of the GLP samples, y-axis represented tumor suppressor genes.

**Figure S2.EBV signature.** Expression of pre-defined EBV signatures in GLP patients

**Figure S3. GISTIC 2.0 amplifications** **and deletions in 2 CNV group. A-B** Chromosomal locations of peaks of significantly recurring focal amplifications and deletions are plotted.

**Figure S4.EMT signature.** Expression of pre-defined EMT signatures in GLP patients


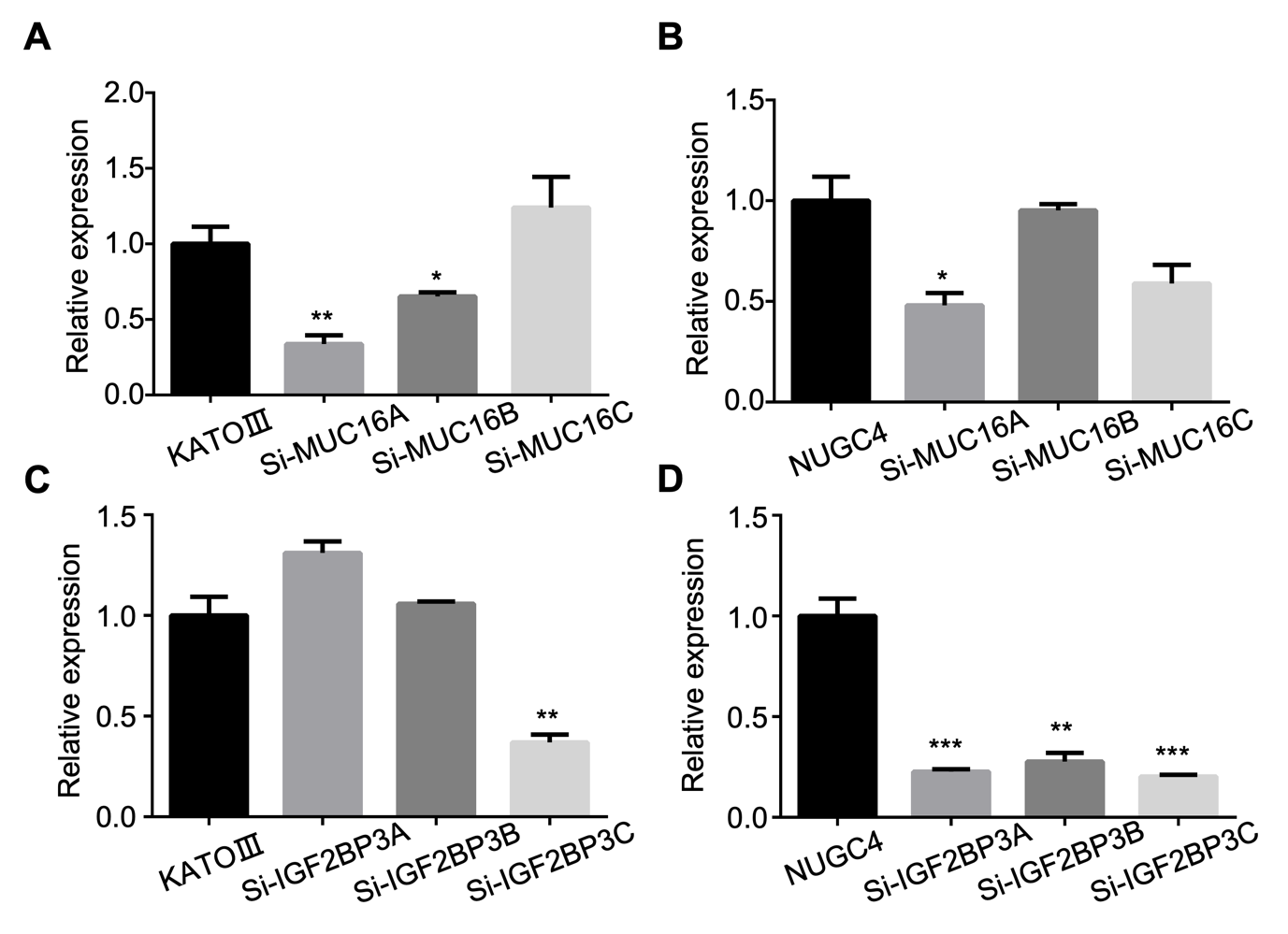


**Figure S5. Knockout validation. A-B** qRT-PCR analysis of MUC16 mRNA levels in untreated and MUC16 siRNA-transfected **A** KATO III cells and **B** NUGC4 cells. **C-D** IGF2BP3 mRNA levels in **C** KATO III cells and **D** NUGC4 cells.
